# How does hormesis impact biology, toxicology, and medicine?

**DOI:** 10.1038/s41514-017-0013-z

**Published:** 2017-09-15

**Authors:** Edward J. Calabrese, Mark P. Mattson

**Affiliations:** 1 Department of Environmental Health Sciences, Morrill I, N344, University of Massachusetts, Amherst, MA 01003 USA; 2 Laboratory of Neurosciences, National Institute on Aging Intramural Research Program, Baltimore, MD 21224 USA; 3 Department of Neuroscience, Johns Hopkins University School of Medicine, Baltimore, MD 21205 USA

**Keywords:** Neural ageing, Chemical biology

## Abstract

Hormesis refers to adaptive responses of biological systems to moderate environmental or self-imposed challenges through which the system improves its functionality and/or tolerance to more severe challenges. The past two decades have witnessed an expanding recognition of the concept of hormesis, elucidation of its evolutionary foundations, and underlying cellular and molecular mechanisms, and practical applications to improve quality of life. To better inform future basic and applied research, we organized and re-evaluated recent hormesis-related findings with the intent of incorporating new knowledge of biological mechanisms, and providing fundamental insights into the biological, biomedical and risk assessment implications of hormesis. As the literature on hormesis is expanding rapidly into new areas of basic and applied research, it is important to provide refined conceptualization of hormesis to aid in designing and interpreting future studies. Here, we establish a working compartmentalization of hormesis into ten categories that provide an integrated understanding of the biological meaning and applications of hormesis.

## Introduction

In 2002, Calabrese and Baldwin^1^ published a paper entitled “Defining Hormesis”. Since then, rapidly expanding experimental findings about the concept of hormesis have contributed substantially to the better understanding of this concept. The 2002 paper, which contained five independent co-published commentaries/critiques,^2–6^ evaluated hormesis from a relatively broad context by examining its strengths, limitations, and possible applications. That paper can usefully serve as a benchmark as it was published just as hormesis research began its period of accelerated growth. In the year 2000 articles using terms “hormesis” or “hormetic” were cited approximately 400 times, while in 2016 articles using those terms were cited more than 8000 times. Over these years, the knowledge base on hormesis has grown greatly and continues to expand, revealing the need for modification and refinement of the concept. Consequently, this paper examines hormesis in relationship to these more recent research findings, offers insight to and refinement of the concept, and improves clarification of its scientific foundations and biological/biomedical significance. Those interested in a broad overview of the hormesis concept including its historical foundations, biological generality, mechanistic foundations, and environmental and biomedical applications may refer to a series of previous publications.^7–11^

## Refinement #1: Hormesis measures the enhanced performance of multiple integrative biological processes that are each constrained by the limits of plasticity

While hormesis is viewed in the light of evolutionary-based adaptive responses, this concept may also be seen as a measure of performance and resilience of any living system including, for example: cell proliferation, fecundity, cell and tissue repair, disease resistance, behavioral/cognitive endpoints, aging/longevity and others that are fundamental for survival and thriving in challenging environments.^8, 12^ Hormesis represents a central evolutionary strategy that is constrained by the limits of biological plasticity. The fact that such integrative and adaptive responses share similar quantitative features broadly across phyla suggests that a key evolutionary compromise was adopted between the degree to which biological performance (i.e., amplitude of stimulation) occurs and the cost of such enhanced performance within the context of managing limited biological resources. Based on the enormity and diversity of hormetic responses across the plant, microbe and animal kingdoms, the consistently modest stimulatory range of hormesis (between 30 and 60% above controls) represents and actually defines the limits of biological plasticity.^13, 14^ Hormesis is also characterized by the simultaneous stimulation of many independent cellular functions/endpoints—each with its own set of quantitatively hormetic features (such as enhancements of DNA repair, antioxidant defenses, autophagy, and others)—whose actions are regulated by multiple interacting receptor/signaling pathways that ultimately produce a metabolically integrated and coherent cellular response. In other words, hormesis is a coordinated response of cells and organisms to an imposed or intrinsically generated challenge that involves multiple integrative signal-transduction processes, each of which is quantitatively hormetic, to coordinate a final holistic response.

## Refinement #2: Hormesis is fundamental to evolution and highly generalizable

Within the context of hormesis, “generalizability” refers to the large numbers of independently derived hormetic observations that have been reported across all animal and plant phyla. The fact that hormesis is often produced in response to stimulatory processes and across all forms of life strongly suggests that its origins are evolutionary and highly conserved. Luckey was an early and strong proponent^15, 16^ of this notion that Calabrese and Blain,^17^ and Mattson^18^ would later significantly strengthen and document over the ensuing decades.

**Fig. 1:**
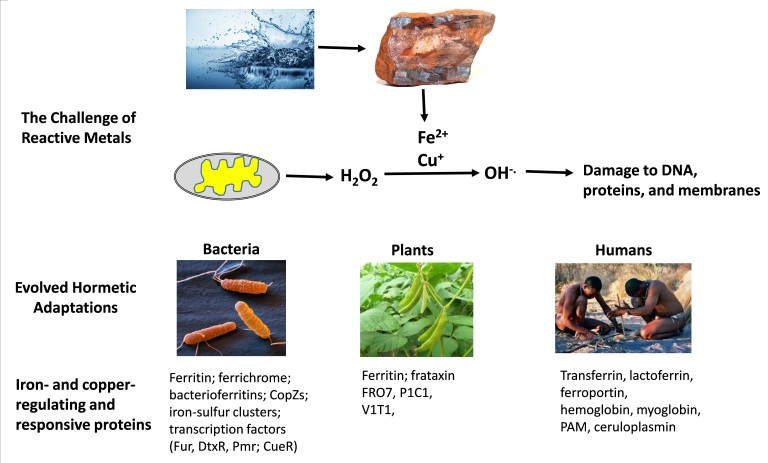
Evolutionary hormesis-based adaptations that enabled organisms to survive and flourish in the presence of toxic metals. The solubilization of iron and copper in rocks results in the formation of ions (Fe^2+^ and Cu^+^) that can be highly toxic to cells. During respiration (oxidative phosphorylation), cells generate hydrogen peroxide (H_2_O_2_). Interaction of H_2_O_2_ with Fe^2+^ or Cu^+^ results in the generation of the highly destructive hydroxyl free radical (OH^−.^), which can kill cells by damaging DNA, proteins, and membrane lipids. Beginning very early in the evolution of life, organisms evolved proteins to protect themselves against Fe^2+^ or Cu^+^ toxicity. The proteins include those that sequester the metal ions or expel them from the cell. In addition, various iron- or copper-dependent enzymes evolved that used the redox properties of these elements to their advantage. Examples of proteins involved in iron and copper metabolism are shown. *FRO7* ferric chelate reductase oxidase 7; *P1C1* permease in chloroplasts; *PAM* peptidylglycine-alpha-amidating monooxygenase; *V1T1* vacuolar iron transporter 1. All images in the figure were obtained from Wikimedia Commons under the Creative Commons copyright 4.0 International, 2.0 Generic, and Share Alike 2.5 Generic (CC-BY) license, and GNU Free Documentation 1.2 license

One remarkable example of how cells and organisms evolved to survive exposures to toxic agents and, moreover, to use those toxic agents to their advantage concerns the metals iron and copper (Fig. 1). Iron and copper leached from rocks are present in oceans, lakes, streams, and aquafers in their ionic forms, Fe^2+^ and Cu^+^. These metal ions can be toxic to cells because they can trigger the production of highly reactive free radicals that damage and kill cells. Thus, even the most primitive cells had evolved multiple mechanisms to protect against iron and copper.^19, 20^ Bacteria produce several different iron and copper-binding proteins and, even evolved several enzymes that require either iron or copper to function properly (e.g., cytochrome oxidases, superoxide dismutase 1, and multicopper oxidases). As plants evolved, an ability to tolerate iron and copper enabled them to increase their distribution into areas with high concentrations of these metals in the soil.^21^ Iron and copper regulation in mammals is remarkably complex, involving concentrations of certain metal-binding proteins in specific types of cells (e.g., hemoglobin in red blood cells and myoglobin in muscle cells), and chaperone and transport proteins that shuttle the metals from the blood into various organs.^22^ In humans, the ‘hormetic zones’ for iron and copper have been established, and dysregulation of iron and copper homeostasis are involved in a wide range of diseases including neurodegenerative disorders such as Parkinson’s disease.^23^

The concept of hormesis is far more expansive and generalizable than would normally be inferred when using only the terms hormesis and hormetic in searching large databases such as Pub Med and Web of Science. Many other terms are frequently used to report hormetic responses in the scientific literature, including the Arndt-Schulz Law, biphasic dose response, U-shaped dose response, preconditioning/adaptive response, overcompensation responses, rebound effect, repeat bout effect, steeling effect, among others. As a result, scientists searching for studies on hormesis frequently overlook a vast reservoir of many relevant publications, which would appear to diminish the broader generalizability of the concept. Unpublished findings by Calabrese and colleagues estimate that using only hormesis or hormetic as the search terms with PubMed or Web of Science could result in overlooking upward of 80–90% of the articles that may satisfy entry and evaluative criteria for inclusion in the hormesis database. In reality, therefore, a substantial, expansive and diverse amount of basic, applied and mechanistic research exists on the topic of hormesis.

This estimation was not only surprising but also provided part of the motivation and rationale to integrate within the framework of hormesis a number of concepts and terms related to biological stress.^24^ Including these relevant stress terms as apt descriptors of a hormetic response greatly enhances and generalizes the scope and importance of hormesis.

## Refinement #3: The frequency of hormesis in the biological and biomedical literature

Several studies have estimated the frequency at which hormesis occurs in the biological, toxicological, and pharmacological databases. Such estimates are very challenging to achieve because of the difficulty in detecting a hormetic dose response that is (1) small-to-modest in size and/or (2) unpredictable or variable in its time of occurrence. Ideally, to identify the optimal hormetic dose response, a large dosing range involving many samples and multiple experiments would be required. Then, to prevent missing the time window of the hormetic response, multiple kinetic (dose–time) experiments would also be required. That toxic doses could elicit responses on a time scale different than hormetic doses could further complicate the process and require extra dose–response and dose–time experiments. And since most dose–response studies in the literature were never designed as hormetic studies, it would be quite unlikely that the dose–response and dose–time experiments of these same literature studies would be predisposed to show a hormetic response. The capacity to detect hormesis, therefore, is significantly affected by the quality of the study design, the number of doses/concentrations, the dose spacing in the low dose zone and the statistical power of the study. This suggests that there is a high likelihood that a certain proportion of potentially bona fide hormetic dose responses may not be detectable with rigorous *a priori* entry and evaluative criteria. Nonetheless, estimates of hormetic dose response frequency using such a priori entry and evaluative criteria have approached 40%.^25–29^ It is recognized that any estimate of hormesis frequency will have some limitations and/or disagreements concerning journal selection, years reviewed, biological models, endpoints, and chemicals assessed, as well as selection of a priori entry and evaluative criteria, among other factors. However, such potential limitations are tempered by a strong consistency of findings across multiple studies using different models and a priori entry and evaluative criteria.

## Refinement #4: Quantitative features of hormetic dose responses are independent of hormetic mechanisms

During the 1980s and 1990s, an important and widely perceived criticism of hormesis was its paucity of explanatory mechanisms. One early and discerning respondent to such criticisms was Szabadi,^30^ who wisely pointed out that the mechanisms of many “biphasic” dose–responses of potentially beneficial drug candidates—especially those involving integrated receptor-mediated pathways—were already present in the pharmacological literature. Szabadi’s own research on “biphasic” mechanisms was in fact broadly expansive and widely supported by the pharmacology community.^31–33^ In sharp contrast, research classified as specifically “hormetic” was published primarily in the toxicological literature and focused on so-called anomalous, stimulatory effects produced by low-doses of potentially harmful agents. Because “hormetic” (unlike “biphasic”) publications were uncommon, the concept of hormesis was not widely disseminated and consequently received very little support from the toxicology community. In effect, the terms “biphasic” and “hormetic” were each associated with a distinct scientific discipline and were generally considered to be two separate and distinct forms of biological responses. It, therefore, should not be too surprising that the mechanistic references of Szabadi to the “biphasic” responses of pharmacological agents were neither recognized nor accepted by toxicologists as plausible explanations for the “hormetic” responses of toxic agents. This situation, however, profoundly changed over the past decade as the literature on hormesis greatly expanded with detailed characterizations of many examples of specific molecular and cellular hormetic signaling mechanisms (for reviews see refs. 9, 34–37).

While the biological and biomedical communities were demanding mechanisms to account for the observation of hormetic-like (biphasic) dose responses, so too were the regulatory agencies, such as the United States Environmental Protection Agency (EPA), demanding modes of action/mechanisms for all agents that were to be regulated. The need to provide mechanistic understandings for the actions of regulated agents is considered an essential requirement because it assists in predicting toxic outcomes and has risk assessment implications. Of particular significance is that the quantitative features of hormetic dose responses were shown to be independent of mechanism.^9^ Thus, while the EPA finds mechanistic information to be of importance in the risk assessment of traditional modes of toxicity, this does not appear to be true in the case of hormesis where maximum responses appear to be limited by the constraints of biological plasticity. It is a significant and novel discovery that the quantitative features of hormesis—regardless of model, endpoint and inducing agent—are not affected by mechanism.

## Refinement #5: Pre-, post-, and remote-conditioning and adaptive responses are manifestations of hormesis

**Fig. 2:**
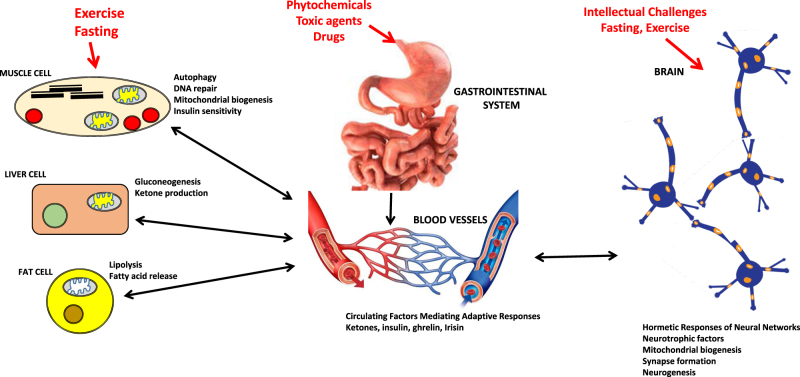
Responses to hormetic challenges are coordinated across multiple organ systems, and involve both cell autonomous molecular mechanisms, and signals transmitted between different tissues. Exercise and fasting impose bioenergetic challenges to multiple organ systems, with responses of muscle, nerve cell networks, liver, and adipose cells being particularly important during the exercise. A major source of exposures to potentially toxic agents is their ingestion as components of food and water, or as man-made drugs. Numerous signaling molecules are released into the blood in response to environmental challenges, and function to coordinate hormetic responses of various organ systems. The brain plays major roles in adaptive responses to a wide range of hormetic exposures, mediating both immediate responses, and enduring changes in synaptic connectivity, and learning and memory, that optimize performance under challenging environmental conditions. (for in-depth discussion see refs. 36, 41, 77). Images for Digestive Tract and Capillaries from Wikimedia Commons under the Creative Commons copyright (CC-BY-SA) 2.5 license, other images in Fig. 2 were created by author M Mattson and have not been previously published

The concept of preconditioning originated from studies investigating the effects of radiation on plant growth and go as far back as the late 1920s (see ref. 10 for a review). Nearly 50 years later, radiation was again employed, this time by Wolf and colleagues^38^ to demonstrate what is now well known as the adaptive response. Essentially, they showed that low doses of ionizing radiation stimulated protective cellular adaptations that significantly reduced the rate at which mutations were produced by a subsequent higher dose of radiation. A little later, in 1986, Murray^39^ showed that preconditioning the heart with ischemic stress protected it against (reduced) damage from a subsequent heart attack. The later study and many others have played an important role in initiating and expanding research areas related to both preconditioning and adaptive responses, especially with respect to generalizability (applicable to numerous stressors or endpoints), biomolecular mechanisms and practical applications. Particularly prominent has been research advances in metabolic hormesis in relation not only to ischemic preconditioning of the heart and brain, but also to the health and therapeutic benefits of exercise and fasting for the body and brain^40–43^ (Fig. 2). Toward the important goal of developing applications, however, research also needs to be conducted on learning how to optimize the conditioning period (i.e., the reliability, sustainability, and scalability) of these protective responses. Furthermore, a sizable number of observations from a recent study indicated that these conditioning and adaptive doses are exactly the same as hormetic doses.^44^ That is, when different conditioning doses were each compared to the size of the protective response that each had elicited following the application of a high challenging (damaging) dose, the protective responses conformed to not only the quantitative features but also the general profiles that have been shown to characterize a hormetic dose response.^10, 44^ These observations have essentially integrated pre-and post-conditioning and adaptive responses into the expansive conceptual framework known as hormesis.^18, 24^

Extensive documentation has shown that preconditioning and the adaptive response are manifestations of hormetic dose responses.^10, 44^ Importantly, recent findings from experiments using animal models, are demonstrating that hormesis can occur when a challenge is imposed after acute injury or the onset of a chronic disease has occurred. Examples of such “postconditioning” include postinjury metabolic challenges (e.g., moderate ischemia, exercise, and fasting) in stroke, myocardial infarction, and traumatic tissue injuries and surgery.^45–48^ Moreover, hormetic signals emanating from a tissue under stress can be communicated to distant tissues, a general phenomenon referred to as “remote conditioning”.^49^ A better understanding of the cellular and molecular mechanisms of pre- post- and remote-conditioning is leading to the development of novel interventions to promote optimal health and resilience.

Mushak^50, 51^ suggested that the hormetic process requires too much time to respond effectively to a toxic threat. He stated that the pre-hormesis time lag basically means hormesis delayed, and hormesis delayed is hormesis denied. Such a characterization of the temporal sequence is misleading when one considers that there are both early (1 h) and late (12–72 h) periods of protection.^10, 44^ These preconditioning processes are part of an adaptive evolutionary strategy for dealing with environmental challenges. They indicate that biological systems evolved early as well as late-phase responses to help ensure survival from acute as well as chronic threats, respectively. Furthermore, since post-conditioning hormesis also protects against quick-acting potentially fatal insults this supports both the presence and effectiveness of hormetic processes that are evolutionarily-based. Of special importance is the recognition that these responses are hormetic and, as such, they are complex, integrated and energetic processes that demand prompt and efficient re-allocations of biological resources to provide protection against all kinds of unexpected stressors (agents and conditions) in a timely fashion.^10, 44^

## Refinement #6: Hormesis has quantitative features that are similar in normal and high risk groups, enabling it to inform drug development

The concept of hormesis has enabled the development of numerous pharmaceutical agents. A detailed consideration of multiple areas in the development of pharmaceuticals reveals biphasic (hormetic) dose responses of many drugs. For example, biphasic dose responses of anxiolytic^52^ and anti-seizure^53^ drugs have been frequently reported during various stages of preclinical studies.^11^ The hormetic data from these studies were used to select doses for testing during clinical trials. Hormesis has played a critical role not only in the development of drugs by the pharmaceutical industry but also in the evaluation of pharmaceutical data by the Food and Drug Administration.

Of particular importance in the assessment of pharmaceuticals and related agents by regulatory agencies is whether the hormetic concept can be applied to both normal and high-risk groups. Calabrese and Baldwin^54^ explicitly addressed this issue using information within the Hormesis Data Base. The analysis revealed that both normal and high-risk groups typically display hormetic dose responses to the same inducing agent. While the quantitative features of the hormetic dose responses were similar between the normal and high-risk groups, the latter groups tended to respond at a lower dose, that is, the dose response was shifted to the left. The capacity for hormetic dose responses to occur in normal and high-risk groups provides not only challenges for the clinical trial but also unique opportunities, within the context of personalized medicine to exploit inter-individual variations and target the pharmaceuticals to select subgroups

## Refinement #7: Integration of linear no threshold (LNT) and hormesis to optimize (cancer) risk assessment

Recent studies have shown that doses corresponding to the estimation of cancer risk at 10^−4^ on a LNT dose–response model are the same as the doses corresponding to the estimation of cancer risk at the nadir on the J-shaped hormetic dose-response model. This observation provided the basis for the recommendation that the essential features of LNT and hormesis could integrate into a public health-based model that optimizes carcinogen risk assessment for the public.^55, 56^ This integrated dose–response represents a model uncertainty approach for cancer risk assessment, with the LNT providing an upper bound for uncertainty and the hormetic model providing a lower bound for uncertainty. Thus, the dose corresponding to a cancer risk of 10^−4^ on the LNT model represents the dose where the maximal estimation of public health benefit would occur on the hormesis model. Deviations in either direction would reduce estimated optimal health benefits (i.e., increasing doses would increase cancer risk and decreasing doses would decrease hormetic health benefits). While this integrated dose–response approach has been proposed for cancer risk assessment, it might also apply to other disorders. For example, physiological or pharmacological challenges that induce metabolic and oxidative stress within a certain range can protect the brain in animal models of Parkinson’s and Alzheimer’s disease.^41, 57^

## Refinement #8: Hormetic synergy

The concept of chemical interaction, including additivity and synergy, is well established in toxicology. However, for hormetic dose responses, this concept is strikingly different. Even though the data are limited, the findings suggest that maximal responses in the hormetic stimulatory zone are still constrained to the 30–60% increase in amplitude. However, additive and synergic effects can occur at low response levels as studied in the case of memory enhancing drugs.^58^ Stimulatory responses from such hormetic interactions become progressively less effective as the 30–60% ceiling response is approached. This concept is relevant in both clinical pharmacology for drug–drug interactions and in environmental toxicity for assessments of contaminants, such as endocrine disrupting agents. It is also consistent with the notion that organisms have evolved to coordinate their cellular and molecular responses to multiple environmental challenges so as not to overreact to an extent that is detrimental.

## Refinement #9: Temporal hormesis—extending the resilient phenotype

Hormesis-mediated resilient phenotypes have typically been considered to be a transitory phenomenon, perhaps lasting several days to about a week. However, it would be of considerable biomedical significance if resilient phenotypes could be extended for a prolonged period. In this regard Gidday^59, 60^ has been able to extend the resilient phenotype of several days to 6–8 weeks in rodent glaucoma and stroke models. Other research groups have also been successful with respect to extending the duration of the resilient phenotype.^61–63^ Park et al.^61^ reported that the protection induced via ischemia preconditioning in the BALB/c mouse kidney remained quite significant even after 12 weeks. A prominent example of a transient exposure resulting in an enduring adaptive response is an emotional challenge that results in a lifelong memory of that experience. Formation and retention of such memories can be considered a hormetic response, with the involved nerve cell circuits being subjected to excitatory stress that signals enduring structural and biochemical changes in the synaptic connections of those neurons.^64, 65^

Interestingly, a case for extending the resilient phenotype transgenerationally via hormetic mechanisms has been reported by Kishimoto.^66^ Using *C. elegans* they reported that hormetic effects induced in the parental generation can be inherited. When the parental *C. elegans* was exposed to a wide range of stressors during developmental stages enhanced resistance to both oxidative stress and proteotoxicity was observed. These adaptations were passed on to subsequent generations via epigenetic mechanisms even when grown in unstressed conditions. These findings reveal a cross-generational communication strategy, which provides the offspring with survival advantages for dealing within a range environmental changes.

## Refinement #10: Hormesis: overestimating stimulation and underestimating frequency

**Fig. 3:**
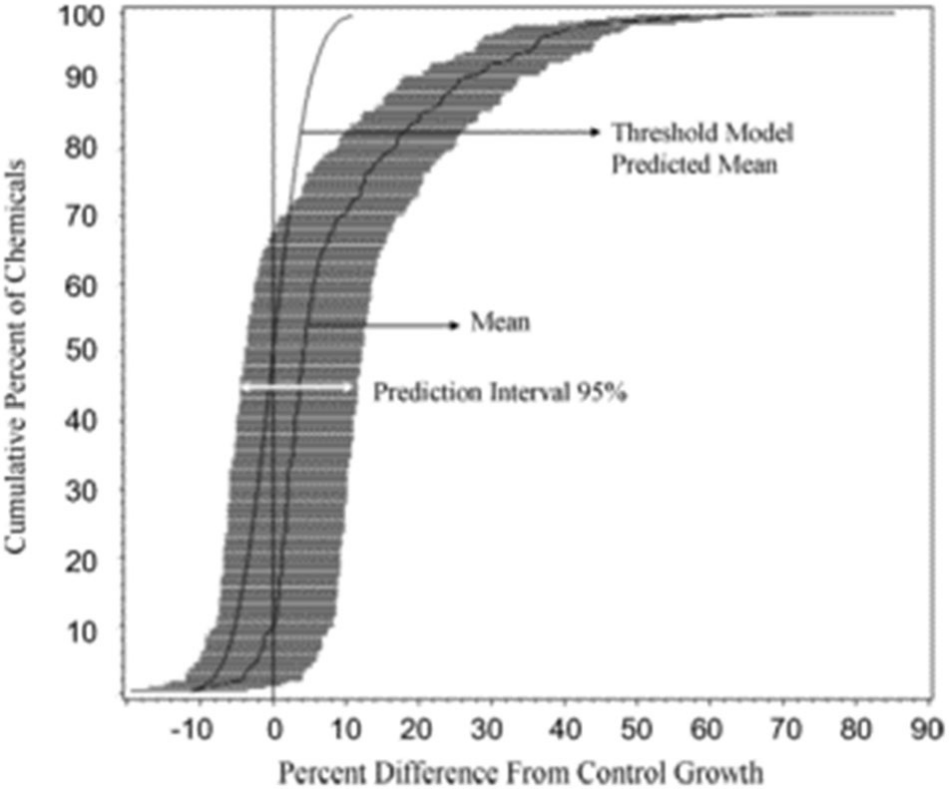
Distribution of predicted mean response and 95% prediction interval values of the 253 chemicals satisfying the a priori entry criteria for the wild-type strain with three responses below the BMD_5_. These findings are compared to expectations for a threshold model (Source: ref. 28).

If a threshold dose response accounted for responses below the estimated threshold, then the responses below the threshold would be randomly distributed with an average value equal to the control group (100%). Figure 3 (Threshold Model Predicted Mean) represents the simulated distribution (i.e., based on known response variability) of control responses of a large number of chemicals (i.e., 253), showing the symmetry of the random responses above and below the average value.^29^ Thus, there are responses greater than and less than the control average, but when combined they equal 100%. Figure 3 also represents a distribution of hormetic responses (i.e., treatment group responses below the threshold dose, showing the mean and the prediction interval 95% across the entire distribution of agents tested). The entire distribution is shifted to the right of the simulated control group chemical response distribution, indicating a stimulatory treatment group response across the entire distribution of agents tested, even the less stimulatory agents. Thus, while these “low” responding agents would be generally viewed as non-hormetic (i.e., not stimulatory), this distributional response analysis reveals that the treatment responses are greater than those of the control group responses at the same location within the distribution response of agents. This suggests that essentially all the chemicals tested are showing hormesis but that standard hypothesis statistical approaches would usually only detect chemicals shifted further to the right, such as those in the upper right quadrant. These findings suggest that efforts to estimate the frequency of hormesis significantly underestimate the actual value. In contrast to this underestimation of hormetic frequency, the findings suggest that the magnitude of the response in the upper right quadrant would need to be adjusted downward to the degree that the response is shifted to the right for the controls. Thus, in this case the responses are somewhat exaggerated when compared to a zeroed out control value. This analysis illustrates that the estimates of the maximum stimulation of hormesis has the potential to be overstated while the frequency of hormesis would be understated (Table 1).

**Table 1:** Hormesis conceptual refinements since the year 2000

No.	
1	Hormesis as a measure of biological performance for integrative endpoints.
2	Biological performance that is enhanced via hormesis is constrained by the limits of plasticity.
3	Hormesis is highly generalizable.
4	Hormesis is frequent in the biological/biomedical literature.
5	Hormetic mechanisms are now extensively documented.
6	Quantitative features of hormetic dose responses are independent of hormetic mechanisms.
7	Pre-, post-, and remote-conditioning are manifestations of hormesis.
8	Hormesis has a key role in drug development.
9	Hormesis is similar in normal and high-risk groups.
10	Integration of LNT and hormesis may optimize cancer risk assessment.
11	Hormetic synergy occurs within evolutionarily constrained limits.
12	Transient hormetic challenges can result in extended resilience.
13	An explanation of why the maximum stimulation of hormesis is over estimated.
14	An explanation of why the frequency of hormesis in the literature is underestimated

## Concluding remarks

The hormesis concept has become well established in the biological and biomedical literature for chemicals, pharmaceuticals, ionizing, and non-ionizing radiation, and physiological challenges such as exercise and food deprivation. This development resulted, in part, from resurgence of interest in radiation and chemical hormesis in the mid to late 1970s primarily under the leadership of Luckey and Stebbing, respectively. A growing interest led to the first hormesis conference in 1985 and the publication of its proceedings in Health Physics in 1987. Another important factor in the evolution of this concept was the highly visible commentaries on the topic in the journal *Science* by Leonard Sagan^67^ and Sheldon Wolff,^68^ whose laboratory discovered the adaptive response in radiation.^38^ Continuing progress in the assessment of hormesis would occur throughout the 1990s under the leadership of BELLE (Biological Effects of Low Level Exposures) at the University of Massachusetts at Amherst, whose efforts would result in nearly regular annual conferences on hormesis,^69–71^ the creation of a widely distributed scholarly newsletter on hormesis and the eventual creation of a professional journal (i.e., dose–response) and a professional society. These efforts would continue to the present, affecting publication of about ten focused books on hormesis and its incorporation into major textbooks on toxicology.

The concept of hormesis would become better understood and refined over this later time period. Among the major refinements was the realization that hormetic dose responses were reproducible and generalizable, being independent of biological model, endpoint and inducing agent. While this was more or less the case by 2000, during the late 1990’s the quantitative features of hormesis were inadequately and less clearly understood. For example, multiple papers published in the late 1990’s^72–75^ used an assessment criteria based in part on the assumption or belief that the magnitude of the hormetic response was substantial, approaching and perhaps even exceeding a response that was fourfold greater than the control response. However, as the Hormesis Data Base continued to expand it became clear that the amplitude of the hormetic stimulation was modest, typically less than twice the control group and usually at a maximum of only 30–60% greater than the control group.^17, 76^ This was a new insight that had significant implications for the role of hormesis in assessing hazard and risk as well as drug efficacy.

This discovery of a maximal mean amplitude of a hormetic response helped elucidate other issues. For example, that the amplitude of a hormetic response was limited to a percent rather than a fold-increase provided a better understanding of hormetic stimulation via both direct or overcompensation processes, as each displayed similar quantitative features via differing mechanisms. This discovery also suggested that the maximal amplitude of a hormetic response reflected the gain within a natural system and represented quantitative limits of adaptability that defined a kind of biological plasticity. Thus, hormesis was not only constrained by plasticity but also described key features of biological plasticity.

During the 1990s to the early 2000s, the most substantial criticism of hormesis was its lack of a mechanistic basis. This criticism has now been effectively addressed as hundreds of hormetic dose–response studies have published mechanisms down to the receptor and cell-signaling pathway.^9^ However, of further importance was the realization that the quantitative features of the hormetic dose response was not affected by its underlying mechanism. That is, the amplitude seems to be controlled by the constraints of plasticity rather than by proximate mechanisms that mediate the biphasic dose response.

The recent studies showing that preconditioning and adaptive responses are manifestations of hormesis are important since these concepts have implications for translational activities in medicine and other biological domains. Within this context it appears that the constraints of plasticity may prevent an enhancement of the amplitude of the hormetic stimulation. However, this does not seem to be the case with respect to limiting the duration over which the resilient phenotype may be extended.^59, 66^ Given the biomedical and clinical significance of enhancing magnitude and extending the duration of the resilient hormetic phenotype, it is expected that efforts will be directed in these directions via metabolic engineering and other molecular approaches.
