# Author Correction: How does hormesis impact biology, toxicology, and medicine?

**DOI:** 10.1038/s41514-023-00101-9

**Published:** 2023-03-31

**Authors:** Edward J. Calabrese, Mark P. Mattson

**Affiliations:** 1 Department of Environmental Health Sciences, Morrill I, N344, University of Massachusetts, Amherst, MA 01003 USA; 2 Laboratory of Neurosciences, National Institute on Aging Intramural Research Program, Baltimore, MD 21224 USA; 3 Department of Neuroscience, Johns Hopkins University School of Medicine, Baltimore, MD 21205 USA

Correction to: *npj Aging* 10.1038/s41514-017-0013-z, published online 15 September 2017

The authors regret that Figure 1 included an image of 2 people trying to start a fire which was labelled as ‘Animals’ instead of ‘Humans’. We sincerely apologize for any offense caused, which was entirely unintentional. Figure 1 has been replaced with a new figure with the correct labels.

